# Competencies in Head and Neck Surgery teaching for medical internship. Position statement of Brazilian Head and Neck Surgery Society^☆^

**DOI:** 10.1016/j.bjorl.2025.101715

**Published:** 2025-09-06

**Authors:** Mario Augusto Ferrari de Castro, Rogerio Aparecido Dedivitis, Fatima Cristina Mendes de Matos, Marianne Yumi Nakai, Leandro Luongo de Matos, Marco Aurelio Vamondes Kulcsar, Flavio Carneiro Hojaij, Carlos Takahiro Chone, Ana Paula Souza, Giovana Meira Muller

**Affiliations:** a Universidade Metropolitana de Santos, Faculdade Medicina, Departamento de Otorrinolaringologia e Cirurgia de Cabeça e Pescoço, Santos, SP, Brazil; b Faculdade de Medicina da Universidade de São Paulo, Departamento de Cirurgia de Cabeça e Pescoço, São Paulo, SP, Brazil; c Universidade Estadual de Pernambuco, Departamento de Cirurgia de Cabeça e Pescoço, Recife, PE, Brazil; d Faculdade de Ciências Médicas da Santa Casa de São Paulo, Departamento de Cirurgia de Cabeça e Pescoço, São Paulo, SP, Brazil; e Faculdade de Medicina da Universidade de São Paulo, Departamento de Anatomia, São Paulo, SP, Brazil; f Universidade de Campinas (UNICAMP), Departamento de Otorrinolaringologia e Cirurgia de Cabeça e Pescoço, Campinas, SP, Brazil; g Universidade Metropolitana de Santos, Faculdade Medicina, Santos, SP, Brazil

**Keywords:** Medical education, Clinical competencies, Internship, Delphi technique

## Abstract

**Objectives:**

To standardize the necessary competencies for medical students related to Head and Neck Surgery.

**Methods:**

The study was performed in 2025. The Delphi process was employed in its sequential phases: the selection of an expert panel; a structured questionnaire containing a preliminary list of potential competencies; the electronic distribution of the questionnaire to the experts, with options to “maintain”, “remove”, or “modify” the competencies and to suggest the inclusion of new competencies; a second round indicating “agree” or “disagree” for each reformulated or new competency; and the final consensus.

**Results:**

Twelve competencies were established for medical students in the specialty of Head and Neck Surgery.

**Conclusion:**

Undergraduate students should be directed toward the most prevalent conditions in the general practitioner's clinical practice.

**Level of evidence:**

4.

## Highlights

• Competency-based education ensures that medical students achieve proficiency.
• An expert panel consisted of 25 specialists participated in Delphi process.
• 12 competencies were generated for medical students in Head and Neck Surgery.

## Introduction

Contemporary medical education faces significant challenges, primarily related to the effective integration of clinical, theoretical, and practical competencies that respond to the dynamic demands of the medical profession. Within this context, the establishment of minimum competencies emerges as a guide in Medical Education, aligning teaching with the real needs of clinical practice. Competencies are tasks or responsibilities that learners must be able to perform independently upon reaching certain levels of proficiency.^1^^,^^2^

In the specific field of Head and Neck Surgery (HNS), the integration of competencies into the educational curriculum is crucial, given the high prevalence of a wide variety of benign and malignant conditions in this region. Medical students must master a series of competencies that go beyond theoretical medical knowledge.^3^

For medical students, early contact with specific competencies, such as recognition of pre-neoplastic lesions and cancer in early stages, specific physical examination, detailed preoperative evaluation, airway management, and basic surgical skills, can establish a solid foundation of clinical competencies. This is corroborated by studies demonstrating that initial exposure to specialized procedures increases students' competence and interest in pursuing specific surgical careers.^4^^,^^5^

The teaching of head and neck surgery in medical schools is quite heterogeneous. Thus, the objective of this study is to formulate the standardization of necessary competencies for medical students related to HNS.

## Methods

This project was approved by the IRB of Universidade Metropolitana de Santos under the number 81479624.2.0000.5509.

This is a qualitative cross-sectional study to determine the competencies related to HNS for medical students at the end of their medical course performed in 2025. Data were collected through electronic questionnaires. The responses received from the questionnaires were organized and evaluated using the Delphi methodology, seeking to reach consensus.^6^

The Delphi process was structured in sequential phases, beginning with the selection of an expert panel comprised of HNS Departments directors affiliated with the Brazilian Society of Head and Neck Surgery, as well the Society’s Board members, ensuring national representation from major training centers.

In the initial phase, a structured questionnaire containing a preliminary list of potential competencies for medical interns in the context of head and neck surgery was developed. This initial compilation was based on a review of national curricular guidelines for medical education. In the second phase, the questionnaire was electronically distributed to the experts, who evaluated each competency using three response options: “maintain”, “remove”, or “modify”. When the “modify” option was selected, participants could suggest specific modifications to the competency in question. Additionally, experts had the opportunity to suggest the inclusion of new competencies they considered relevant that were not included in the initial list.

Following the collection of responses from the first round, qualitative and quantitative analysis of the results was performed, identifying competencies with greater agreement for maintenance, those indicated for exclusion, and those requiring reformulation. Based on this analysis, a second questionnaire was developed, incorporating the suggested modifications and new competencies proposed by the experts. In the third phase (second Delphi round), the revised questionnaire was sent to the same experts, this time requesting them to indicate “agree” or “disagree” for each reformulated or new competency, with space provided for qualitative observations. This dichotomous approach in the second round is consistent with the Delphi methodology when seeking to consolidate consensus after a more comprehensive exploratory first phase.

Final consensus was defined as agreement equal to or greater than 85% after the second round. This rigorous consensus criterion was established to ensure that only truly essential competencies would be included in the final curriculum for medical interns.

The entire process was conducted with anonymity among participants, a fundamental characteristic of the Delphi methodology that minimizes the influence of hierarchical or political factors in decisions.^7^

## Results

The expert panel consisted of 25 specialists who participated in all phases of the Delphi process. The first round comprised the proposition of 12 competencies. Six competencies were unanimously approved, six had suggestions for modifications, and there were no suggestions to withdraw any of the proposed competencies. Three new competencies were also suggested. After generating the new listing, the second-round analysis showed 100% agreement on seven competencies, 95% on 4 competencies, and 90% on 1 competency, resulting in 12 competencies ‒ Table 1.

**Table 1:** Medical internship competencies in head and neck surgery.

**#**	Competency
1	Act with ethics and professionalism
2	Communicate gently, effectively, and empathetically with patients, family members, and other professionals
3	Work appropriately in a team
4	Conduct thorough medical history for patients with head and neck conditions
5	Perform physical examination of patients
6	Identify the main risk factors for head and neck cancer
7	Identify conditions requiring immediate airway management
8	Identify thyroid gland disorders
9	Identify the most prevalent head and neck conditions
10	Identify conditions requiring biopsy
11	Perform primary skin closure
12	Perform postoperative dressing changes

## Discussion

Competency-based education aims at standardized levels of proficiency, with the objective of ensuring that all students achieve a certain level of proficiency upon completion of training.^8^ Therefore, competencies can be considered skills or capabilities and are the organizing units of the competency-based medical curriculum.^9^

The population incidence of head and neck conditions is high, making basic airway management and recognition of risk factors for cancer in this anatomical region important. Most head and neck tumors are diagnosed at advanced stages. Many patients experience significant delays in diagnosis and treatment because they require multiple consultations before reaching a specialist. Additionally, some general healthcare professionals fail to recognize certain signs and symptoms as suggestive of malignancy.^10^ Part of the undergraduate workload allocated to HNS should be utilized in practical teaching activities, such as proper loco-regional examination and training in suspecting risk factors for malignancy among patients with habits such as smoking, symptoms like persistent dysphonia, and signs such as oral ulcers that do not heal for more than three weeks.

Situations that are not part of the primary level of care, such as details of staging and treatment options for various head and neck tumor sites and complaints that require specific examinations, will be experienced at other levels of professional training. The consensus established in this research proposes global knowledge in highly prevalent contents of the specialty, enabling the general practitioner to suspect the diagnosis and appropriately refer the patient, preventing important conditions from being underdiagnosed.

## Conclusion

Through consensus, students should be directed and trained for the topics and procedures frequently encountered in general practice, which should focus on the most prevalent conditions. Time should be dedicated to theoretical and practical learning, aiming to achieve higher levels of competence in the most common conditions.

## ORCID ID

Mario Augusto Ferrari de Castro: 0000-0003-0234-9807

Fatima Cristina Mendes de Matos: 0000-0002-9135-8117

Leandro Luongo de Matos: 0000-0002-5068-8208

Marco Aurelio Vamondes Kulcsar: 0000-0002-4751-0476

Flavio Carneiro Hojaij: 0000-0003-0080-022x

Carlos Takahiro Chone: 0000-0002-4217-4629

Ana Paula Souza: 0009-0001-5582-9356

Giovana Meira Muller: 0000-0002-2839-8255

## Funding

None.

## Declaration of competing interest

The authors declare no conflicts of interest.
